# Advancements in the Application of CVD Diamond Detectors for Space Radiation Monitoring

**DOI:** 10.3390/nano16181176

**Published:** 2026-09-17

**Authors:** Qingwen Meng, Guohong Shen, Zhe Yang, Donghui Hou, Huanxin Zhang, Zida Quan, Ying Sun

**Affiliations:** 1 National Space Science Center, Chinese Academy of Sciences, Beijing 100190, China; 2 Beijing Key Laboratory of Space Environment Exploration, Beijing 100190, China; 3 University of Chinese Academy of Sciences, Beijing 100049, China

**Keywords:** space environment detection, diamond detector, chemical vapor deposition, high-energy particles, space applications

## Abstract

With advancements in space exploration, the extreme conditions of space—such as ionizing radiation, temperature gradients, and micrometeoroid impacts—pose significant challenges to the operational stability and longevity of space-based detection systems. Therefore, the development of detectors with enhanced resilience and reliability has become imperative. Recent advancements in synthetic diamond detectors fabricated through chemical vapor deposition (CVD) have demonstrated high radiation tolerance, favorable thermal stability, and reliable radiation-detection performance. These attributes position diamond detectors as promising candidates for space radiation monitoring applications. This study systematically analyzed the physical properties, fabrication methodologies, radiation detection principles, and prospective applications of diamond detectors within the context of space environments, highlighting their advantages in comparison to conventional detection technologies.

## 1. Introduction

Space missions require radiation detectors that can operate reliably under intense ionizing radiation, large temperature variations, and long mission durations. Energetic protons, electrons, heavy ions, and secondary particles can introduce lattice defects, charge trapping, leakage-current increases, and other forms of performance degradation in semiconductor detectors and associated electronic systems [1,2]. These effects are particularly important for long-duration missions and radiation monitoring in harsh environments, where detector stability and calibration reliability directly affect measurement accuracy. Silicon-based detectors are widely used because of their mature fabrication technology and well-established readout ecosystem, but their performance can degrade under high accumulated radiation exposure, motivating the investigation of alternative radiation-hard semiconductor materials [2,3].

Chemical vapor deposition (CVD) diamond is a promising candidate for such applications because of its wide bandgap, low intrinsic carrier concentration, high breakdown field, high carrier mobility, and strong covalent bonding. These properties enable low leakage current, rapid charge collection, and stable operation under high electric fields and elevated radiation levels [3,4]. However, the practical advantages of diamond must be considered together with several limitations. High-quality single-crystal CVD diamond remains relatively expensive, large-area material availability is limited compared with mature silicon technologies, and detector performance can vary with crystal quality, impurity concentration, electrode configuration, and fabrication conditions [5,6]. Current research therefore focuses not only on improving material quality and radiation tolerance, but also on increasing fabrication yield, reducing cost, improving device consistency, and developing scalable detector architectures [7,8,9].

Although several previous reviews have summarized the material properties, growth techniques, and radiation-detection capabilities of diamond, comparatively less attention has been given to linking these topics directly to the requirements of space radiation monitoring. In particular, systematic comparisons between diamond and competing semiconductor detector technologies, distinctions between laboratory demonstrations and in-orbit validation, and practical issues such as detector area, manufacturing scalability, readout integration, calibration stability, and long-term qualification have not always been considered within a unified framework. This review therefore aims to bridge the gap between material-level advances and mission-oriented detector assessment.

This review primarily considers developments reported from 2000 to 2026, while selected earlier studies are retained where they provide essential background or foundational detector concepts. The scope includes the intrinsic properties and CVD growth techniques of diamond, radiation-interaction and signal-formation mechanisms, representative detector architectures, and applications to charged-particle, neutron, and ultraviolet/vacuum-ultraviolet detection. The review is organized around four principal questions: (1) which material and device characteristics make CVD diamond competitive with Si, GaAs, and GaN for space radiation detection; (2) how crystal quality, growth conditions, detector architecture, and electrode configuration affect measurable detector performance; (3) which reported results represent laboratory proof-of-concept, space-oriented ground qualification, or demonstrated in-orbit operation; and (4) what material, manufacturing, integration, calibration, and reliability barriers still limit broader space deployment. By addressing these questions, this review seeks to provide a mission-oriented assessment of both the advantages and the remaining limitations of CVD diamond radiation detectors.

## 2. CVD Diamond

### 2.1. Advantages of Diamond in Radiation Detection

The wide bandgap of diamond (5.5 eV), together with its extremely low intrinsic carrier concentration (<10^3^ cm^−3^) and high resistivity (>10^16^ Ω·cm), contributes to very low leakage current and favorable signal-to-noise performance [10]. Under an applied voltage of 1000 V, a 500 μm thick diamond detector produces a dark current of only 30–40 pA/cm^2^, allowing it to maintain high sensitivity at elevated voltages, making it suitable for high-precision measurements. The dielectric constant of diamond is 5.7, lower than that of silicon (11.7), which results in reduced input capacitance in the detector’s readout circuit. This lower capacitance minimizes capacitance-induced noise currents, effectively suppressing noise and enhancing detection performance [10]. Additionally, diamond exhibits high electron and hole mobilities of approximately 4500 and 3800 cm^2^/V·s, respectively, enabling efficient carrier transport and rapid charge collection. Combined with its low leakage current and high breakdown field, these properties support fast signal response, high-field operation, and radiation detection under demanding environmental conditions [11,12,13].

Microwave plasma chemical vapor deposition (MPCVD) enables the production of large, high-quality diamond films. MPCVD facilitates the growth of high-quality diamond films with improved size and reproducibility, thereby expanding the practical applicability of diamond detectors [14]. Diamond’s exceptional melting point, ranging from 3550 to 4000 °C, and its low thermal expansion coefficient (0.8 × 10^−6^/K) contribute to its robustness under extreme conditions. Chemically inert, diamond resists corrosion from acids and alkalis, further enhancing its suitability for harsh environments. The strong sp^3^-hybridized C–C bonding contributes to the high displacement energy and radiation tolerance of diamond. Compared with conventional silicon detectors, diamond devices can retain useful charge-collection performance at high irradiation fluences, although the degree of degradation depends on particle type, fluence, detector structure, and operating conditions [15]. Table 1 summarizes representative room-temperature material properties of diamond, Si, GaAs, and GaN that are particularly relevant to radiation-detector performance. These values provide a material-level basis for comparison, although parameters such as carrier mobility and breakdown field may vary with material quality and measurement conditions.

Although the intrinsic material properties summarized in Table 1 provide the physical basis for detector performance, these parameters alone do not fully represent the practical capabilities of radiation detectors. For space applications, experimentally demonstrated charge-collection performance, radiation tolerance, leakage current, high-temperature operation, and space-related validation are also important considerations. Therefore, Table 2 further compares representative quantitative performance metrics of diamond, Si, GaAs, and GaN radiation detectors. Because the reported values were obtained using different detector structures, radiation sources, irradiation conditions, bias voltages, and operating temperatures, they should be regarded as representative experimental benchmarks rather than strict one-to-one comparisons.

The comparison indicates that no single semiconductor detector technology is superior in all performance metrics. Silicon benefits from highly mature wafer-scale fabrication, extensive space heritage, and established readout technologies, whereas GaAs provides attractive room-temperature spectroscopic performance and GaN combines a wide bandgap with promising high-temperature and radiation-tolerant operation. Diamond is particularly competitive in applications requiring very low leakage current, high charge-collection efficiency, high radiation tolerance, and elevated-temperature operation. However, its higher material cost, limited large-area high-quality CVD availability, and device-to-device uniformity remain important obstacles to widespread deployment. Therefore, detector selection for space missions should be based on the specific radiation environment, mission duration, sensitive area, thermal conditions, and system-level constraints rather than on a single material parameter [27].

### 2.2. Key CVD Techniques for Diamond Preparation

CVD diamond films have been investigated for a broad range of applications, including radiation detection and dosimetry, optical systems, thermal management, high-power electronics, and nuclear-fusion technologies. For radiation-detection applications, the ability to tailor material quality, thickness, impurity concentration, and device structure during CVD growth is particularly important. However, the scarcity and high cost of natural diamond limit its use in high-quality, large-scale applications. Furthermore, natural diamonds often exhibit variations in size, morphology, and crystal quality, while obtaining large-area substrates with uniform properties remains challenging. These limitations hinder their application in large-scale radiation detector fabrication and have driven extensive research into synthetic diamond production. Since the 1950s, synthetic diamonds have increasingly replaced natural diamonds. High-pressure high-temperature (HPHT) and chemical vapor deposition (CVD) are currently the two most widely employed methods for diamond synthesis. However, HPHT is generally limited to the production of small-sized diamond crystals because it requires specialized equipment to maintain extremely high pressure and temperature conditions. The growth size and crystal quality are restricted by the limited volume of the high-pressure cell and the difficulty in maintaining uniform temperature and pressure distributions during synthesis [28]. In addition, HPHT-grown diamonds often contain higher concentrations of nitrogen and metal impurities introduced from the catalyst–solvent system, which may degrade their optical and electrical properties [29,30]. In contrast, CVD offers greater operational flexibility, minimizes impurity incorporation through precise control of reaction gas composition and flow rate, and enables the synthesis of large-sized, high-quality diamonds [28]. Consequently, CVD has become the dominant technology for diamond fabrication. Several CVD techniques are currently in use, including hot-filament chemical vapor deposition (HFCVD), DC plasma-assisted chemical vapor deposition (DC-PACVD), DC arc plasma jet chemical vapor deposition (DC Arc Jet CVD), and microwave plasma chemical vapor deposition (MPCVD) [31,32,33].

The choice of CVD process affects detector performance primarily through its influence on impurity incorporation, defect density, residual stress, and spatial uniformity. These material characteristics affect carrier trapping, mobility, and lifetime and therefore directly influence charge-collection distance and charge-collection efficiency. In particular, nitrogen- and silicon-related impurities, dislocations, grain boundaries, and other extended defects can introduce trapping centers and degrade carrier transport. HFCVD and DC-based processes may suffer from contamination associated with heated filaments or electrodes, whereas MPCVD provides an electrodeless and relatively stable plasma environment that facilitates improved control of impurity incorporation and material uniformity. For large-area growth, maintaining uniform plasma density, substrate temperature, impurity concentration, and growth rate across the deposition surface is also essential. Therefore, the suitability of a CVD technique for radiation-detector fabrication should be evaluated not only in terms of growth rate and deposition scale, but also in terms of defect density, impurity concentration, carrier transport, and process reproducibility.

**Figure 1:**
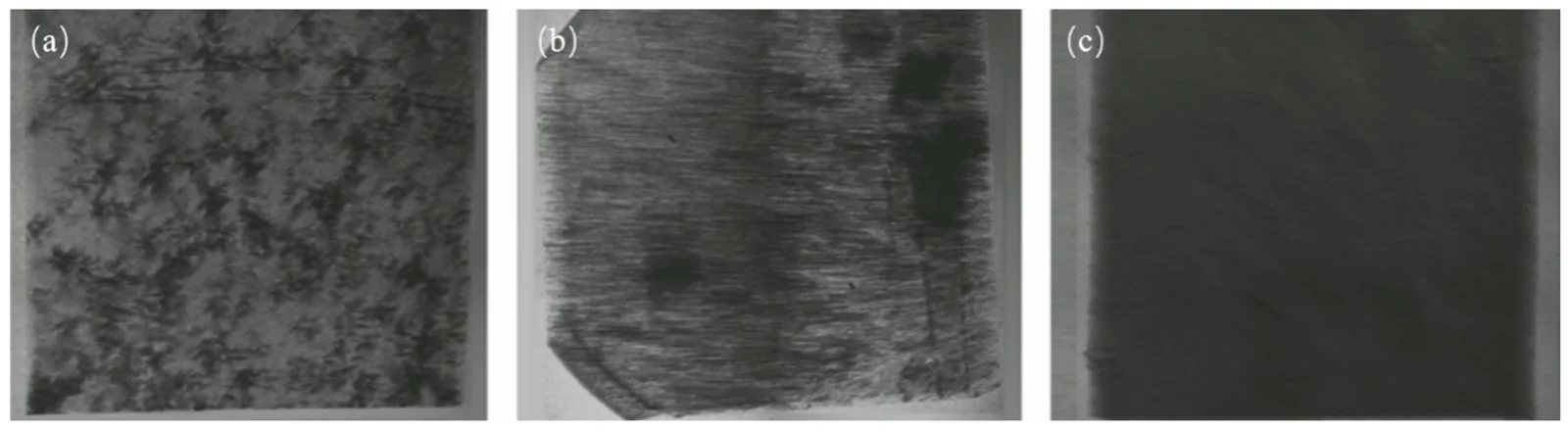
X-ray white beam topography of high-purity single crystal diamond, slightly nitrogen-doped single crystal diamond, and reference sample [34]: (**a**) high-purity monocrystalline diamond, (**b**) slightly nitrogen-doped monocrystalline diamond, (**c**) reference sample. Adapted with permission from Ref. [34]. Copyright 2020, the copyright owner.

**Figure 2:**
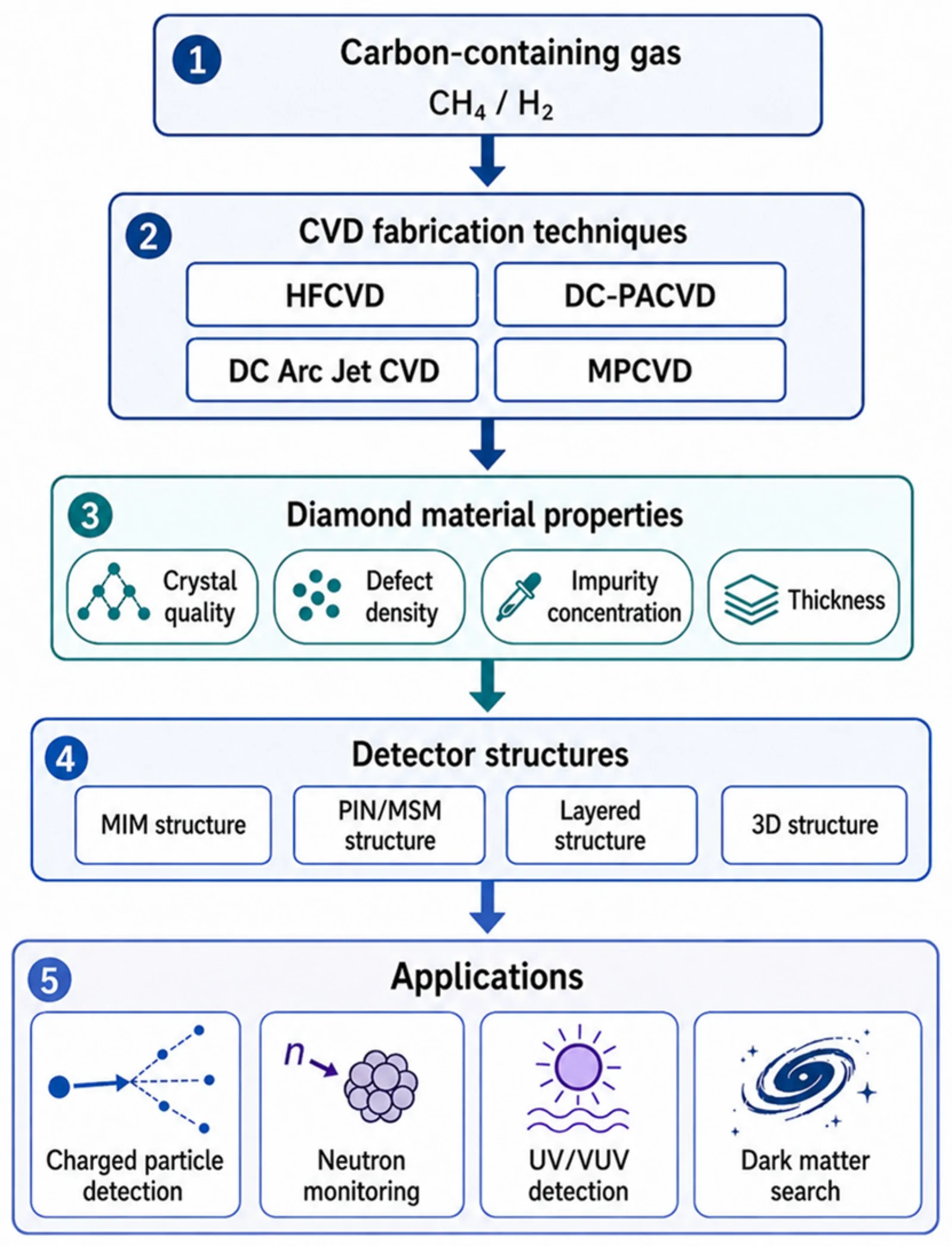
Schematic illustration of CVD diamond fabrication and application pathways.

Table 3 summarizes the key features of these methods. Among these techniques, MPCVD is widely used for detector-grade diamond growth because it provides stable plasma conditions, relatively low contamination, and good control over deposition parameters. These characteristics are favorable for reducing impurity incorporation and structural defects and for improving the reproducibility of carrier-transport properties across the grown material. By controlling trace impurity levels, Li et al. [34] successfully reduced defects in diamond and enhanced its overall performance. The resulting monocrystalline diamond exhibited a uniformly distributed dislocation stress field and fewer internal defects. Its transmittance in the ultraviolet to near-infrared wavelength range reached 71.58%, closely approaching the theoretical transmittance of diamond (71.6%). Additionally, nitrogen-doped monocrystalline diamonds achieved a comparable transmittance of 71.27%, demonstrating optical performance similar to that of high-purity monocrystalline diamonds. X-ray white-beam topography provides a nondestructive means of visualizing lattice defects, dislocations, and their associated stress fields in single-crystal diamond. As shown in Figure 1, the high-purity single-crystal diamond exhibits relatively localized and uniformly distributed dislocation stress fields, whereas the slightly nitrogen-doped sample shows more extended and approximately linear stress distributions. The reference sample presents a much darker topographic contrast over a large portion of the crystal, indicating a higher density of extended defects and lattice distortion [34]. These observations demonstrate that X-ray white-beam topography is useful for evaluating crystal quality and the spatial uniformity of structural defects. Since defect and impurity distributions are closely related to carrier transport and charge trapping in diamond, such structural characterization is also relevant to assessing the suitability of CVD diamond materials for radiation-detector fabrication. Figure 1 therefore provides a direct comparison of the internal structural quality of the three diamond samples, while Table 3 summarizes the characteristics of the four CVD techniques. Figure 2 summarizes the relationship between CVD fabrication techniques, diamond material properties, detector structures, and their corresponding application fields [35,36].

### 2.3. Principles of Particle Radiation Detection Using Diamond Detectors

#### 2.3.1. Energy Deposition and Carrier Generation

When ionizing radiation traverses a diamond sensor, it deposits energy in the lattice and creates electron–hole (e-h) pairs along its track. For minimum-ionizing charged particles (MIPs), the energy loss in diamond is nearly constant over a broad energy range, and the typical ionization yield is of the order of 35–40 e-h pairs per micrometer of path length in high-purity material [36]. The average energy required to create one e-h pair in diamond is about 13 eV, significantly higher than in silicon. Combined with a wide bandgap of 5.5 eV, this leads to a very low intrinsic carrier concentration and negligible dark current even at room temperature.

As a consequence, although diamond generates fewer charge carriers per unit dose than silicon, it can be operated at relatively high electric fields while maintaining low leakage current, which is beneficial for signal-to-noise performance and operation in high-radiation environments such as particle accelerators and space [41,42].

#### 2.3.2. Signal Formation and the Ramo–Shockley Theorem

The formation of current pulses in diamond detectors is rigorously described by the Ramo–Shockley theorem, which applies to any solid-state radiation detector. For a carrier of charge q moving with instantaneous velocity $\vec{v}(t)$, the current induced on a given electrode is

(1) $$i(t)=q\vec{v}(t)\cdot {\vec{E}}_{w},$$

where ${\vec{E}}_{W}$ is the so-called weighting field associated with that electrode, obtained by solving Laplace’s equation with unit potential on the readout electrode and all other electrodes grounded [43]. The total current is the superposition of contributions from all generated carriers.

In a typical planar device, metal contacts are deposited on the two opposite faces of a diamond plate, and a uniform electric field $\vec{E}$ is established between them. When the top electrode is biased at a positive potential relative to the bottom electrode, electrons drift towards and are collected by the positive electrode (anode), while holes drift towards the negative electrode (cathode). Their motion in the weighting field induces a transient current; a charge-sensitive preamplifier integrates this current to produce a voltage pulse whose amplitude is proportional to the induced charge and, under limited trapping, to the energy deposited in the active volume [41,44].

#### 2.3.3. Charge Collection Distance and Charge Collection Efficiency

A key figure of merit for diamond sensors is the charge collection distance (CCD), defined as

(2) $$CCD=\frac{Q_{collected}}{Q_{ionized}}\;L,$$

where L is the detector thickness, Q_ionized_ is the charge initially released by ionization, and Q_collected_ is the charge measured at the electrodes. In a simple drift model, the CCD can be approximated as

(3) $$CCD\approx (\mu_{e}\tau_{e}{+\mu }_{h}\tau_{h})\;E,$$

where μ_e,h_ and τ_e,h_ represent the mobilities and lifetimes of electrons and holes, respectively, and E is the applied electric field. This approximation is based on a simplified carrier drift model, in which the charge transport process is dominated by carrier drift under a uniform electric field. The effects of carrier trapping are incorporated through the carrier lifetime parameters, while complex factors such as spatially varying electric fields and non-uniform defect distributions are neglected. This simplified model provides an effective description of charge transport behavior in diamond detectors.

#### 2.3.4. Interaction Mechanisms for Different Radiation Types

Because diamond is a low-Z, wide-bandgap semiconductor, its detection response strongly depends on the interaction mechanism and energy of the incident radiation. The dominant processes differ among charged particles, photons, and neutrons, as discussed below.

##### Charged Particles

Charged particles interact with diamond predominantly through Coulomb interactions with electrons and nuclei. Heavy ions, low-energy protons and α-particles can be fully stopped in typical detector thicknesses, depositing all their kinetic energy and producing large ionization signals suitable for spectroscopy. High-quality single-crystal devices achieve energy resolutions at the level of a few tenths of a percent for α-particles and around 1% for 14 MeV neutron-induced charged secondaries in optimized systems [42,45].

For minimum-ionizing particles such as relativistic pions or protons, only a fraction of the particle energy is deposited; nevertheless, CVD diamond strip and pixel detectors have demonstrated efficient tracking and radiation hardness in accelerator environments with fluences up to ~10^15^ cm^−2^ [41]. These results are directly relevant to space missions exposed to intense proton and heavy-ion fluxes in radiation belts and solar particle events.

##### Photons (X/γ) and Deep-UV/VUV Radiation

X-rays and γ-rays interact with diamond via the photoelectric effect, Compton scattering and pair production, depending on photon energy. The relatively low atomic number of carbon implies modest interaction probabilities for MeV γ-rays in thin layers, but thicker crystals and converter layers can be used to enhance detection efficiency where required [46]. For continuous X-ray fields and high cumulative doses, diamond dosimeters have demonstrated good linearity and long-term stability, with reported operation up to total doses of the order of 10 MGy [42].

In the deep-UV and vacuum-UV (VUV) range, the wide bandgap of diamond results in intrinsic “solar blindness”: photons with wavelengths longer than about 220–230 nm cannot excite carriers across the bandgap, leading to negligible response in the visible and near-IR. Diamond photoconductors and Schottky photodiodes therefore provide highly selective detection of solar EUV/VUV radiation with extremely low dark current and visible rejection factors up to 10^6^, making them attractive for space-borne solar monitors [44,45,46,47,48].

##### Neutrons

Neutrons are detected indirectly via nuclear interactions with carbon or with converter nuclei. In pure diamond, low- and intermediate-energy neutrons interact mainly through elastic scattering on carbon nuclei, producing recoil carbon ions that deposit energy in the lattice and generate detectable ionization. The probability of such interactions depends on neutron energy and thickness; for 1 MeV neutrons in a 0.5 mm diamond, only a small fraction undergo any interaction, and an even smaller fraction produce recoils above the electronic noise threshold.

At higher neutron energies, threshold reactions involving carbon, such as ^12^C(n,α)^9^Be, become important and produce energetic charged particles that can be detected through their ionization in diamond. For thermal and epithermal neutrons, pure diamond is essentially insensitive, and thin converter layers based on 6Li or 10B (e.g., LiF or boron-containing films) are commonly deposited on the detector surface to generate charged reaction products; diamond detectors with LiF or boron converters have been demonstrated for thermal neutron dosimetry and spectrometry, achieving detection efficiencies of a few percent and stable operation at high flux [45].

These interaction mechanisms enable CVD diamond to be used for charged-particle, photon, and neutron detection. Together with its demonstrated radiation tolerance, these characteristics support its consideration for long-term radiation-monitoring applications in space.

## 3. Space Applications of CVD Diamond

The radiation environment encountered by a spaceborne detector depends strongly on orbit and mission type. In low-Earth orbit (LEO), the radiation field is dominated by trapped protons and electrons in the terrestrial radiation belts, particularly during passages through the South Atlantic Anomaly, together with contributions from solar energetic particles (SEPs) and galactic cosmic rays (GCRs). Representative trapped-particle energies extend from tens of keV for electrons to tens or hundreds of MeV for protons, while GCR particles typically extend from hundreds of MeV to GeV per nucleon. In geostationary Earth orbit (GEO), energetic electrons in the outer radiation belt are particularly important and commonly extend from approximately 0.1 to 10 MeV, whereas energetic protons and transient SEP populations also contribute to the radiation environment [49,50,51,52].

Beyond the Earth’s magnetosphere, including lunar and deep-space missions, geomagnetic shielding is largely absent and the radiation environment is increasingly dominated by GCRs and episodic SEP events. GCRs contain predominantly high-energy protons together with α-particles and heavier ions, whereas SEP events can produce intense short-term proton exposures extending from a few MeV to several hundred MeV. On the lunar surface, interactions of primary particles with the regolith additionally generate secondary radiation, including neutrons. These differences among LEO, GEO, lunar, and deep-space environments impose distinct requirements on radiation detectors in terms of detectable energy range, dynamic range, radiation tolerance, particle discrimination, calibration stability, and long-term reliability. Therefore, the relevance of a particular CVD diamond detector should be evaluated against the radiation environment and mission profile for which it is intended.

The studies discussed below represent different levels of space relevance and should therefore not be interpreted as equivalent evidence of technological maturity. In this review, results are distinguished among (i) demonstrated in-orbit operation, (ii) space-oriented ground testing or qualification, and (iii) laboratory proof-of-concept studies. LYRA/PROBA-2 provides direct flight heritage for diamond photodetectors, whereas proton-irradiation studies of detectors intended for future CubeSat missions represent ground-based space qualification. Other accelerator, radioactive-source, and cryogenic experiments primarily demonstrate detector physics or application potential and still require further environmental qualification before space deployment.

### 3.1. Structure of Diamond Sensors

#### 3.1.1. MIM Structure

**Figure 3:**
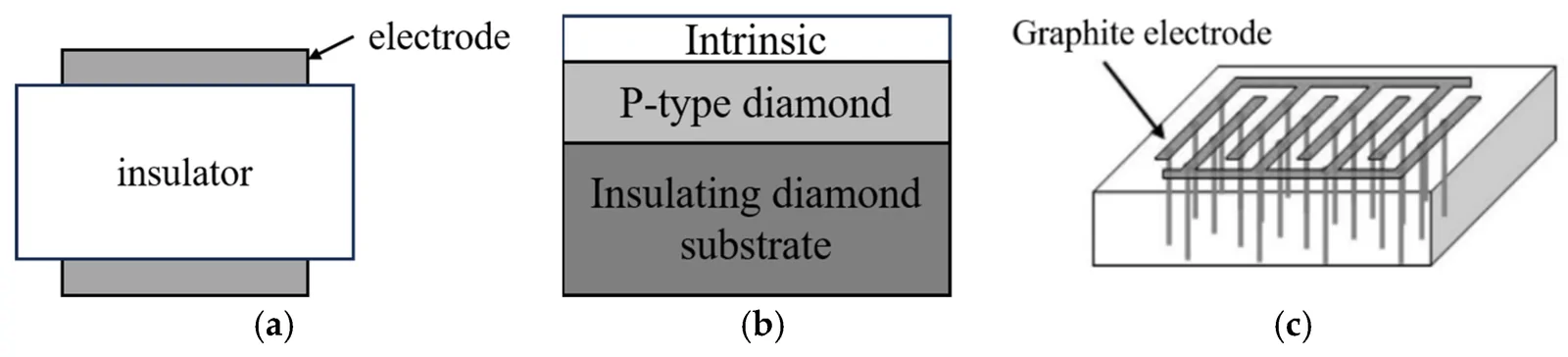
Representative architectures of diamond radiation detectors: (**a**) metal–insulator–metal (MIM) structure, characterized by simple fabrication and straightforward electric-field configuration; (**b**) layered structure incorporating intrinsic and doped diamond layers and rectifying contacts to improve charge collection and spectral response; and (**c**) three-dimensional (3D) structure with buried graphitic electrodes, which shortens the carrier drift distance and enables efficient charge collection at relatively low bias voltages.

The schematic diagram of a metal–insulator–metal (MIM) diamond detector is shown in Figure 3a. A high-resistivity diamond is sandwiched between two metal electrodes. An external voltage applied across the device creates an electric field. Free charges generated by absorbed radiation drift within this electric field, generating a current in the external circuit. The detector can operate in one of two modes: (1) pulse counting mode or (2) current mode. Pulse counting refers to detecting individual quantum events. The output signal may relate to the particle’s energy (spectroscopic mode) or may not (counting mode). In current mode, the detector generates a current proportional to the intensity of the radiation field [53].

#### 3.1.2. Layered Structures

To enhance the energy resolution and manufacturing consistency of diamond radiation detectors, various layered structural designs have been proposed in recent years, as shown in Figure 3b. Kaneko et al. [54] constructed a layered single-crystal diamond detector by growing a boron-doped diamond electrode layer and a 20 μm thick intrinsic single-crystal CVD diamond layer on a HPHT synthesized type Ib diamond substrate. The device integrates boron-doped diamond ohmic contacts and aluminum Schottky contacts, exhibiting pronounced rectifying characteristics. Under irradiation by 5.486 MeV α-particles, the detector achieved energy resolutions of 2.6% and 2.8%, with a rise time of less than 20 ns and a carrier drift velocity exceeding 1 × 10^5^ cm/s, indicating rapid response and high sensitivity. However, the research found detector performance limited by carrier traps and crystal defects, suggesting further improvement in the crystalline quality of the boron-doped layer is necessary.

On the other hand, Almaviva et al. [55] designed a metal/intrinsic/doped p-type diamond sandwich structure Schottky diode detector based on single-crystal CVD diamond. The device has an aluminum top electrode and a boron-doped bottom layer, generating photocurrent under zero bias through the built-in potential barrier. During high-flux X-ray irradiation tests within the 6–20 keV range, the detector demonstrated a linear response and low dark current, consistent with C-V analysis and Monte Carlo simulations. Additionally, the device showed good temporal response and stability, making it suitable for precise soft X-ray detection.

#### 3.1.3. Three-Dimensional Structure

Three-dimensional structured diamond radiation detectors are fabricated by laser irradiation-induced graphite electrodes, providing efficient charge collection at low voltages and improved radiation hardness. Bachmair et al. [56] utilized femtosecond laser-induced phase transformation technology to create graphite/diamond-like composite electrodes in single-crystal CVD diamond, successfully producing the first efficient 3D particle tracking detector, as shown in Figure 3c. This device achieved a most probable charge signal of approximately 13,600 e^−^ during a 120 GeV proton beam experiment at CERN, verifying its feasibility in high-energy physics detection. Lagomarsino et al. [57] constructed 3D diamond detectors using both nanosecond and femtosecond lasers to directly induce graphitized channels in diamond, utilizing Raman imaging to reveal a significant correlation between conductivity and sp^2^ phase content. Electrodes prepared with nanosecond lasers exhibited resistivities as low as 60 mΩ·cm, demonstrating conductivity and compatibility with 3D structures. For three-dimensional diamond detectors with laser-formed buried graphitic electrodes, Salvatori et al. [58] performed a micron-scale mapping of the charge collection efficiency (CCE) using Ion Beam Induced Charge (IBIC) with 3.0 and 4.5 MeV protons. The IBIC maps show that only a few-micrometer-thick radial region around each buried column exhibits a reduced CCE, whereas most of the device volume preserves good detection properties, with a charge collection efficiency of about 90% at a bias voltage of 60 V. In addition, under irradiation with MeV β-particles from a ^90^Sr source, the average collected charge corresponds to a CCE of approximately 96%, confirming that the 3D buried-column geometry ensures almost full charge collection along the detector thickness despite the laser-induced stress and defects around the graphitic pillars.

Overall, these detector architectures involve different trade-offs among fabrication complexity, operating voltage, charge-collection performance, and scalability. Planar MIM detectors offer relatively simple fabrication and mature device processing but generally require the electric field to be applied across the full detector thickness. Layered structures can improve contact characteristics and spectral response through engineered interfaces, although their fabrication requires more precise control of epitaxial growth and doping. Three-dimensional architectures reduce carrier drift distances and can achieve high charge-collection efficiency at relatively low bias voltages; however, their fabrication is more complex, and laser-induced graphitic electrodes may introduce local stress and defects. Therefore, the optimal detector architecture depends on the sensitive area, available bias voltage, power budget, radiation environment, and reliability requirements of the target space mission.

### 3.2. Detection of Charged Particles

**Figure 4:**
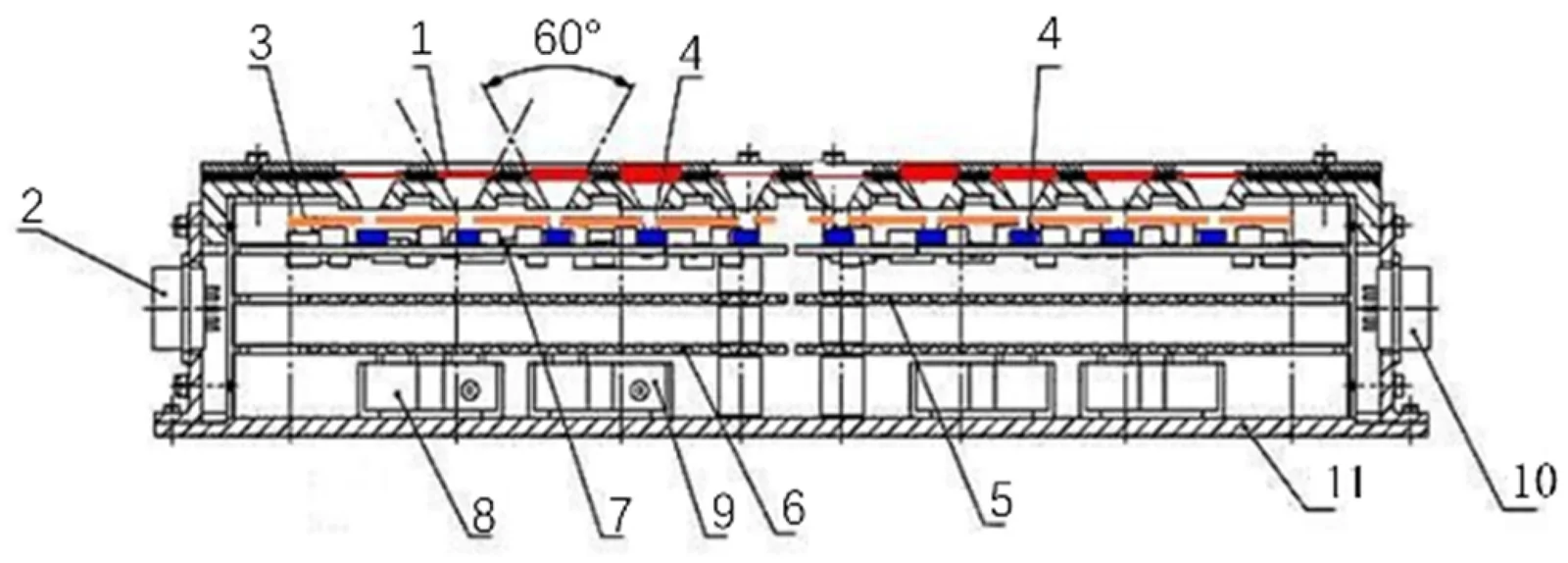
Design of a selective detector for cosmic rays [59]: 1—selective filters, 2—power connector, 3—electrostatic screen, 4—DSE, 5—power supply board assembly, 6—host interface board, 7—detection unit board, 8—filter unit, 9—power supply, 10—connector interface, 11—case. Adapted from Ref. [59].

**Figure 5:**
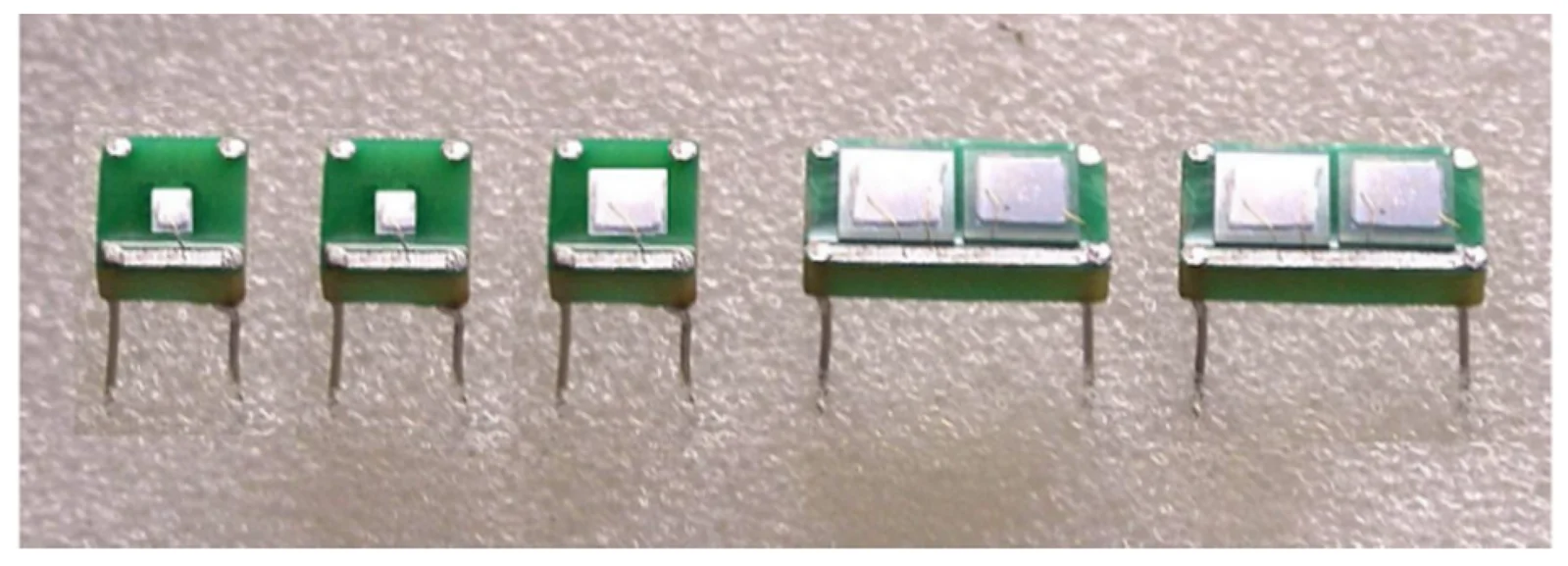
External view of the DSE Adapted from Ref. [59].

In 2015, Altukhov et al. [59] developed a diamond-based detector for selective monitoring of cosmic rays, capable of continuously measuring external electron, proton, and heavy particle fluxes around spacecraft. The detector can measure particle radiation flux density across 12 energy ranges. As depicted in Figure 4, the diamond sensing element (DSE) is mounted on an amplification module situated beneath a collimation window. The collimation window incorporates a selective filter that separates cosmic rays into different energy ranges. The detector employs multiple DSEs with varying sensitive detection surfaces. Figure 5 shows the configuration, where the first two DSEs (surface area: 2 × 2 mm^2^) record the total fluxes of electrons, protons, and heavy particles. The middle DSE (surface area: 3 × 3 mm^2^) detects low-energy protons and electrons with energies above 3 MeV, while the final two DSEs (surface area: 4.5 × 4.5 mm^2^) measure protons and heavy particles with energies exceeding 30 MeV. The design ensures that statistical errors in the measured cosmic particle streams across different energy ranges remain below 10% within a measurement duration of 5–10 min.

**Figure 6:**
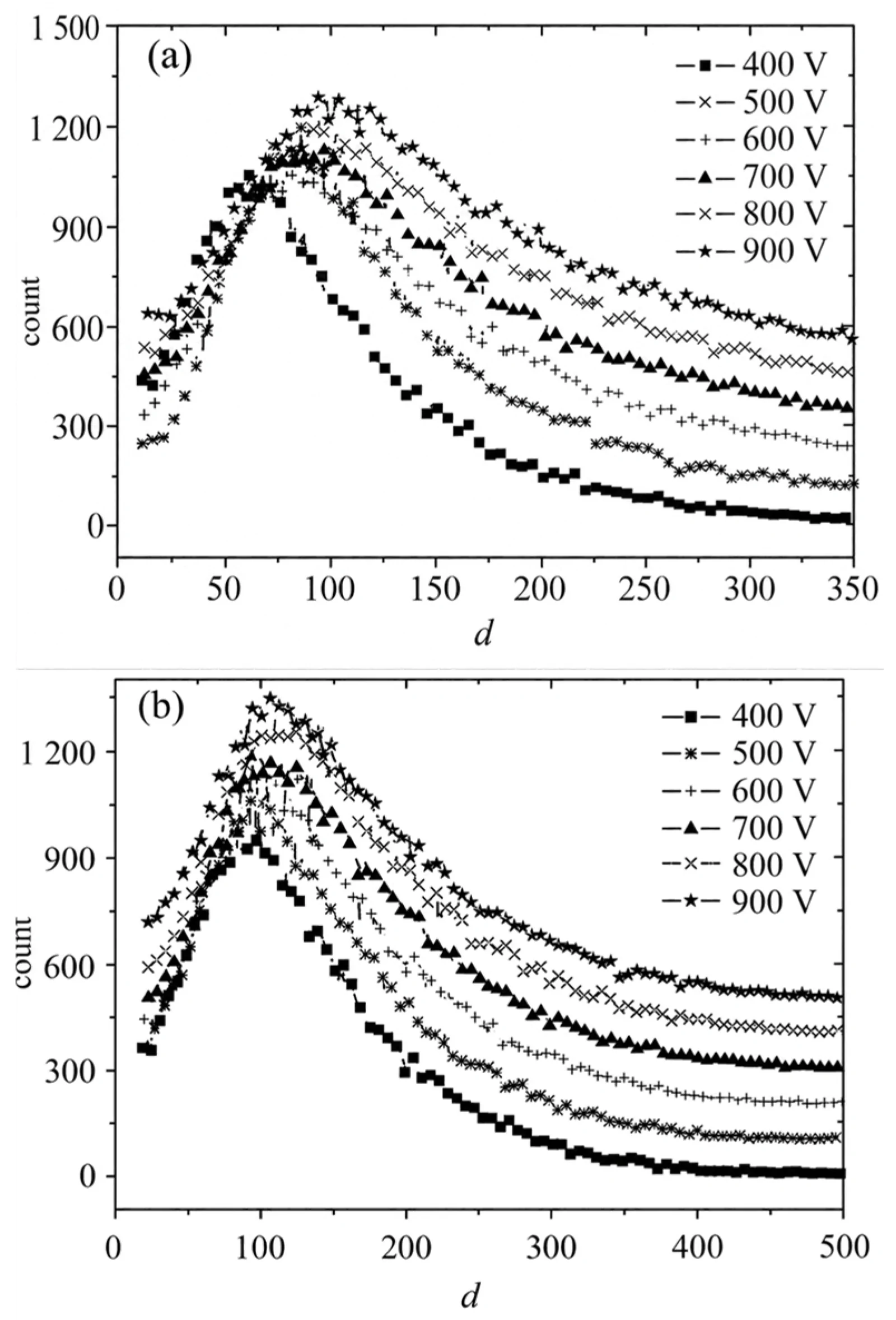
Response spectrum of the diamond detector to 241Am, 243Am, and 244Cm under different bias voltages Adapted from Ref. [61]: (**a**) 241Am source, (**b**) 243Am and 244Cm sources. The horizontal axis represents the multichannel analyzer channel number (d).

Gladchenkov et al. [60] investigated the performance of single-layer and multilayer diamond detectors under various types of ionizing radiation. Their findings indicated that the single-layer diamond detector effectively detects heavy particles but is insensitive to β and γ radiation, as well as neutron radiation with lower LET. They recommended measuring the LET of heavy particles. In comparison, double-layer diamond detectors demonstrated higher charge collection efficiency, the ability to detect low-energy β rays, and a detection efficiency increase of at least 25%. Double-layer diamond film detectors also provided more accurate linear energy transfer density measurements, making them suitable for space particle radiation detection. Furthermore, the use of diamond films with three to five layers enabled the detection of particles in a higher energy range within complex space radiation environments, yielding more precise data. In 2009, Lei et al. [61] utilized a diamond detector with a sandwich electrode structure to test its α-particle spectral response to ^241^Am, ^243^Am, and ^244^Cm sources at 20–25 °C. The results showed the α-particle response spectra at different bias voltages. As depicted in Figure 6, with increasing bias, the charge collection efficiency of the detectors reached saturation. The average CCE was approximately 33.5%, while the relative CCE at the 10% falling edge reached approximately 57%.

**Figure 7:**
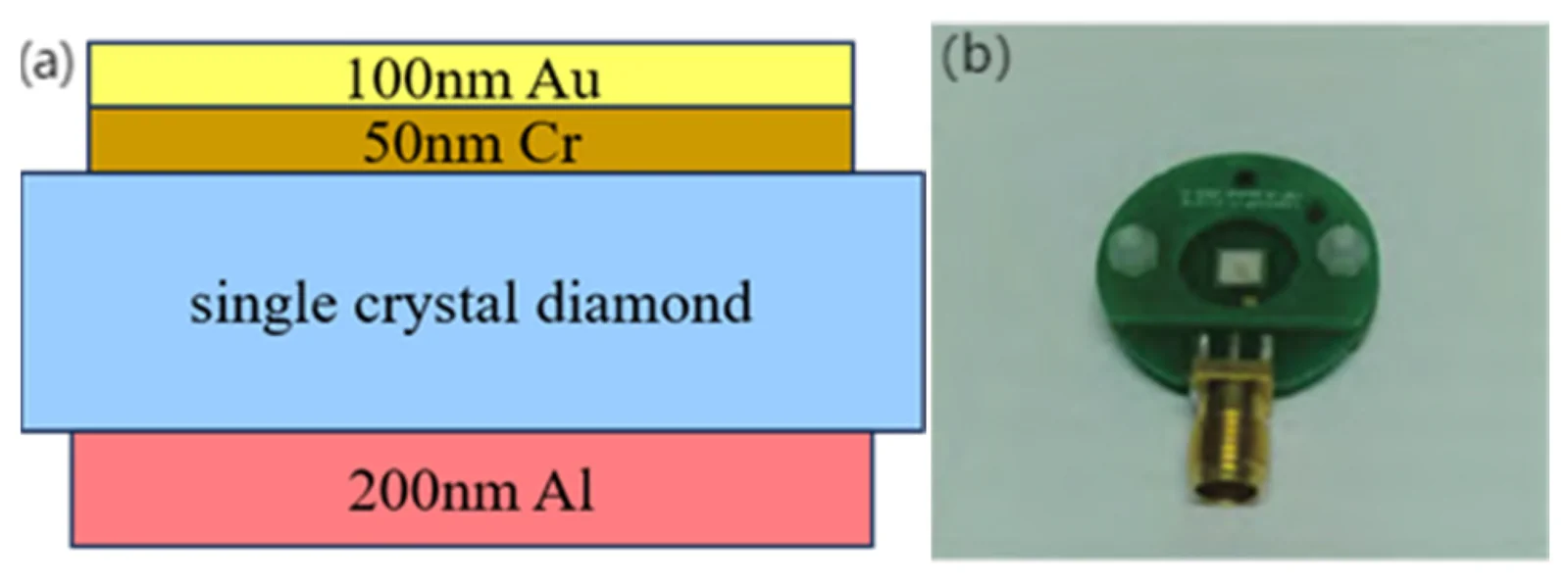
(**a**) Structure and (**b**) physical sample of diamond radiation detector Adapted from Ref. [62].

Liu et al. [62] optimized the SNR, energy resolution, and overall performance of the detectors by modifying the metal electrode structure on the diamond surface. A 4.5 mm × 4.5 mm × 500 μm diamond was used in the study, as shown in Figure 7. The optimized detector exhibited a dark current of less than 0.1 nA at voltages below 100 V, with a charge collection efficiency of 96.9%. The energy resolution was 2.82% at a 380 V bias. These results indicate that electrode configuration and contact engineering can substantially influence leakage current, charge collection, and spectroscopic performance.

Ding et al. [63] employed MPCVD to grow high-quality monocrystalline diamond on a HPHT diamond substrate. By compressing the plasma, they increased the microwave power density, optimized the carbon/hydrogen (C/H) ratio in the plasma, and reduced impurities and dislocations in the diamond epitaxial layer. No impurity bands were detected in the photoluminescence spectra at room temperature. Detectors fabricated using a 200 µm thick epitaxial diamond film exhibited a charge collection efficiency of 97.03% and an energy resolution of 2.1% for electrons, and a charge collection efficiency of 97.86% and an energy resolution of 1.5% for holes. Moreover, the product of the mobility and lifetime of electrons and holes reached 8 × 10^−5^ and 4.1 × 10^−4^ cm^2^/V, respectively.

More recently, Ando et al. [64] investigated two MPCVD diamond radiation detectors developed for space use and evaluated their radiation tolerance using 100 MeV proton irradiation. The detectors are being developed for the KSAT3-X CubeSat mission, which is intended to observe charged-particle populations in the Earth’s magnetosphere. No significant degradation in spectroscopic performance was observed up to irradiation levels corresponding to at least approximately 10 years of the anticipated orbital environment. This result provides ground-based evidence supporting the radiation tolerance of the detector design for the intended space mission, although in-orbit operation has not yet been demonstrated.

Taken together, these studies and recent reviews [65] show that the charged-particle detection performance and radiation tolerance of CVD diamond depend on crystal quality, detector thickness, electrode configuration, contact properties, applied electric field, and irradiation conditions. Earlier CVD diamond film detectors exhibited average charge-collection efficiencies of approximately 33.5%, with values reaching about 57% at the falling edge of the response spectrum [61]. With improved crystal quality and optimized contact structures, substantially higher performance has subsequently been demonstrated. For example, a Schottky-contact diamond detector achieved a CCE of 96.9% with an energy resolution of 2.82% [60], while a high-quality single-crystal CVD detector exhibited electron and hole CCE values of 97.03% and 97.86%, with corresponding energy resolutions of 2.1% and 1.5%, respectively [63]. These results illustrate the strong influence of material quality and device engineering on detector performance. Nevertheless, because the reported values were obtained using different particle sources, detector dimensions, bias voltages, and experimental conditions, they should be regarded as representative benchmarks rather than as direct one-to-one comparisons. For space applications, long-term stability under broad and variable radiation spectra may be as important as the highest CCE or best energy resolution obtained under controlled laboratory conditions.

### 3.3. Neutron Detection

Diamond detectors have been investigated for neutron monitoring because of their radiation tolerance, low leakage current, and capability for detecting charged reaction products. Their neutron response, however, depends strongly on neutron energy and detector configuration, particularly on the use of converter layers for thermal and epithermal neutrons.

In 2018, Holmes et al. [66] investigated diamond PIN diode detectors with a thickness of 4.5 µm and a 0.5 µm BN layer to measure neutron flux distribution in order to accurately assess the space environment. When the detectors were exposed to a thermal neutron beam, the count rate remained roughly constant under 1 MeV neutron irradiation with a 1 MeV neutron-equivalent fluence of 10^15^ n/cm^2^. In 2020, Holmes et al. [67] further evaluated these detectors under a thermal neutron flux of 4.4 × 10^6^ n·cm^−2^·s^−1^. The detector was subsequently irradiated to a 1 MeV neutron-equivalent fluence of 10^15^ n·cm^−2^. After irradiation, the count rate remained nearly unchanged, although the pulse-height distribution shifted to lower values by approximately 250 keV. Measurements with a ^210^Po α-particle source indicated no significant loss of charge-collection efficiency at the operating voltage. These results demonstrate that the tested thin diamond PIN detector retained stable charge-collection performance after substantial neutron irradiation, while the observed pulse-height shift indicates that irradiation-induced changes in the converter layer, contacts, or other device components should also be considered.

For 14 MeV DT neutrons, Shimaoka et al. [68] employed the same single-crystal CVD diamond detectors as compact neutron spectrometers at the Fusion Neutronics Source (FNS), exploiting the ^12^C(n,α)^9^Be reaction as the sensing mechanism. The diamond devices were placed at distances between 30 and 56 cm from the Ti-T target and at observation angles from 95° to 110° with respect to the deuteron beam, and operated under an electric field of about 1.3 V/µm, yielding count rates of up to several hundred counts per second. More recent studies have further investigated CVD diamond detectors for fusion-neutron diagnostics under high-radiation conditions relevant to present and future fusion facilities. These studies have focused on DD and DT neutron spectroscopy, detector lifetime, energy resolution, and operation under elevated temperatures, further demonstrating the potential of single-crystal CVD diamond for fast-neutron measurements in harsh radiation environments [69,70].

**Figure 8:**
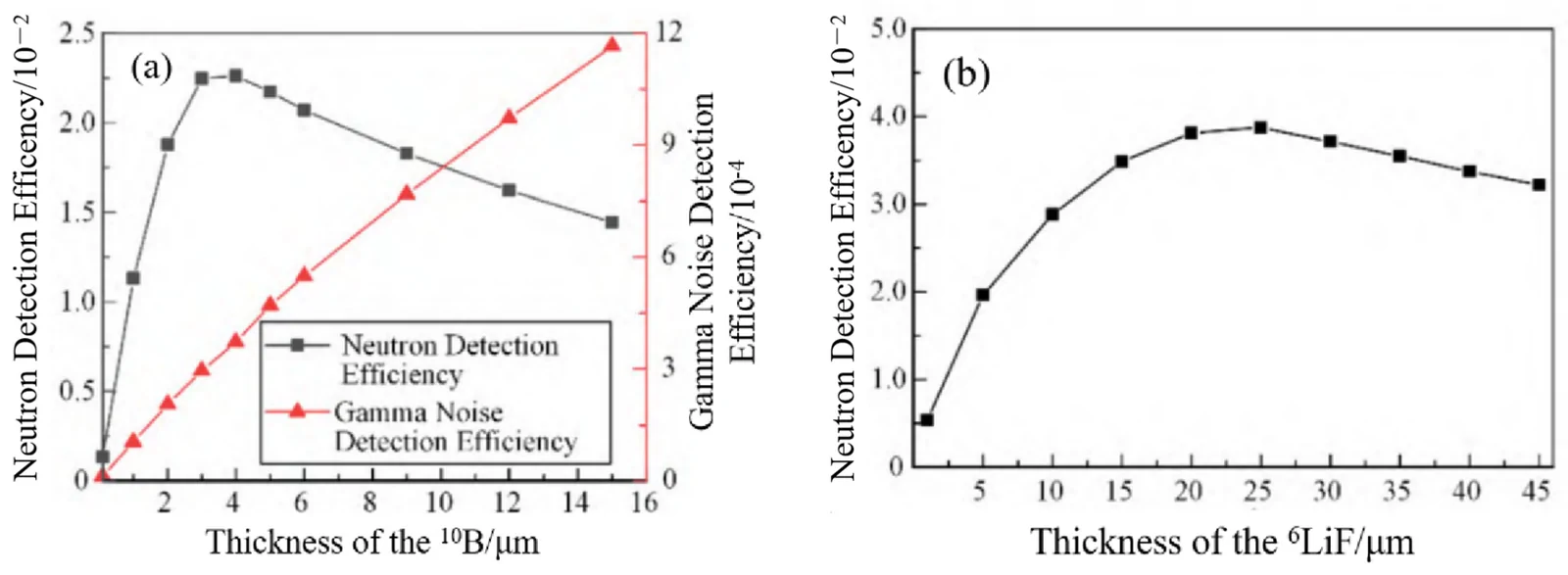
Neutron detection efficiency with different conversion layer thicknesses Adapted from Ref. [69]: (**a**) ^10^B neutron conversion layer, (**b**) 6LiF neutron conversion layer.

In contrast to direct fast-neutron detection through nuclear reactions involving carbon, thermal-neutron detection generally requires a converter layer to generate charged reaction products. Ren et al. [69] employed the MCNP (Monte Carlo N Particle Transport Code) simulation program to construct a physical model of a converter-assisted diamond neutron detector. This study investigated the effects of the neutron conversion layer, diamond film thickness, and γ-screening threshold on neutron detection efficiency. The simulation results revealed a peak in neutron detection efficiency with increasing thickness of the 6LiF conversion layer, which reached a maximum of 3.87% when the ^6^LiF layer was 25 μm thick, as shown in Figure 8.

The reported studies also indicate that the advantages of diamond neutron detectors depend strongly on the neutron-energy range and detector configuration. For slow and thermal neutrons, diamond PIN detectors generally require converter layers such as boron-containing materials or ^6^LiF to generate charged reaction products that can subsequently be detected in the diamond sensitive layer [66,67,68,69]. In such devices, neutron-detection efficiency is constrained by the converter thickness and by self-absorption of the reaction products; consequently, increasing the converter thickness does not necessarily result in a monotonic increase in detection efficiency [67,69]. For example, simulation results using ^6^LiF showed an optimum converter thickness of approximately 25 μm [69]. In contrast, fast-neutron spectroscopy can exploit nuclear reactions directly involving carbon; a 14 MeV neutron spectrometer based on single-crystal CVD diamond achieved an experimental energy resolution of 1.5% and an intrinsic resolution of 0.79% [68]. These results suggest that the principal strengths of diamond neutron detectors lie in their radiation robustness, compact solid-state configuration, spectral capability for fast neutrons, and stable operation in harsh mixed-radiation fields, rather than universally high neutron-detection efficiency.

Beyond neutron spectroscopy, CVD diamond is also being investigated for other fusion-related components that must withstand intense irradiation. Recent studies have examined irradiation-induced defect evolution and recovery in CVD diamond under heavy-ion exposure, as well as the modification of vacancy-related defect centers under proton and carbon irradiation. These investigations are relevant to radiation-resistant diamond-based optical and dielectric components for fusion technologies and further demonstrate that the radiation tolerance of diamond should be evaluated not only in terms of detector signal stability, but also through defect generation, annealing behavior, and changes in functional properties under irradiation [71,72].

### 3.4. Vacuum-Ultraviolet Detection

**Figure 9:**
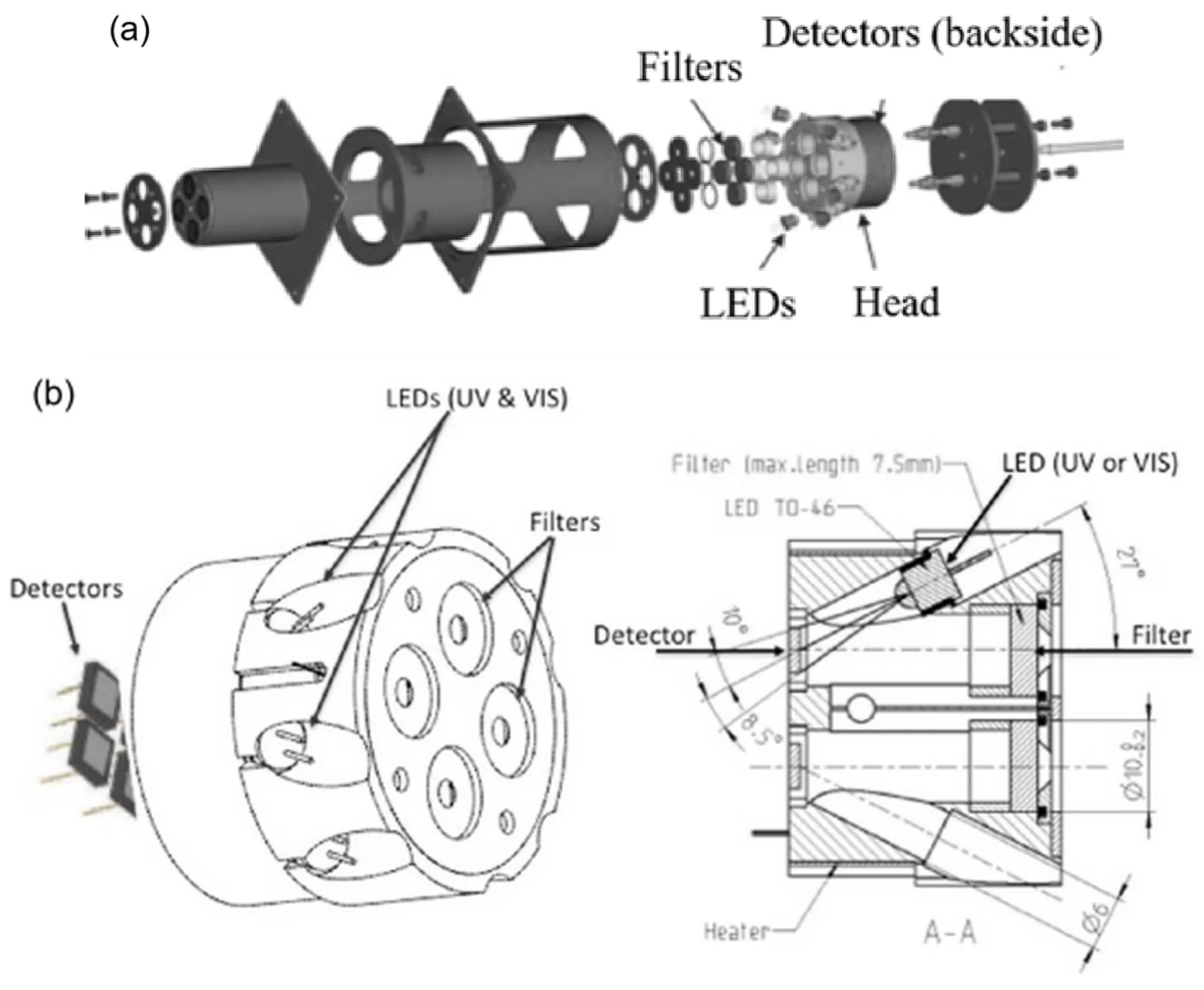
Structural configuration of the LYRA instrument Adapted from Ref. [75]: (**a**) exploded view of one LYRA unit containing four independent spectral channels; (**b**) schematic drawing and cross-section of a LYRA detector head, showing the optical filters, UV/VIS calibration LEDs, detector, and internal optical geometry.

In November 2009, the European Space Agency (ESA) launched PROBA-2 into a sun-synchronous orbit at an altitude of approximately 720 km, carrying the LYRA solar radiometer [73]. LYRA incorporated diamond photodetectors developed by IMOMEC, including devices based on a 0.8 μm thick diamond film epitaxially grown on a type IIa diamond substrate using MPCVD [74]. The instrument comprises three redundant radiometer units, each containing four independent spectral channels. Each channel includes a collimator, precision aperture, spectral filter, photodetector, and UV/VIS light-emitting diodes (LEDs) for calibration. As illustrated in Figure 9, the LYRA channels employ Si-AXUV, PIN diamond, or MSM diamond photodetectors, with the corresponding detector types and spectral bands summarized in Table 4.

The LYRA flight results further demonstrate that long-term detector stability cannot be attributed solely to the intrinsic properties of the detector material. Diamond PIN photodiodes exhibited highly stable dark-current characteristics, with ΔDC values of approximately +0.2 ± 0.2% in all three LYRA units [75]. In contrast, the MSM diamond channels showed substantially larger and channel-dependent variations; for example, the nominally operated Unit 2 exhibited ΔDC values of −32 ± 5%, −10 ± 2%, and −26 ± 5% for different MSM channels [75]. The Si-AXUV reference photodiodes in Unit 3 showed dark-current increases of approximately +13.5–14% after 81 days of cumulative solar exposure [75]. These differences indicate that long-term performance is affected not only by the semiconductor material but also by detector architecture, electrode configuration, operating conditions, and system-level effects. Moreover, the overall LYRA signal degradation should not be interpreted as detector degradation alone, because contaminant accumulation and UV-induced polymerization on the optical filters were identified as major contributors to the observed instrument-level degradation [24,75]. Therefore, LYRA provides important flight heritage for diamond photodetectors while also demonstrating the need to distinguish intrinsic detector stability from the degradation of the complete optical and measurement system.

Recent studies aim to improve light absorption and carrier transport in diamond detectors through new device structures. Liu et al. [76] employed homogeneous epitaxial MPCVD to grow diamond films and developed a 3D diamond detector using a bottom-up process. In this design approach, the integrity of the electrode after growth was influenced by the thickness and adhesion of the tungsten layer on the diamond film. At a voltage of 5 V, these detectors achieved a response rate of 9.9 A/W at 220 nm and demonstrated a rejection ratio of 10^3^ between UV and visible light. The bottom-up process allows for stronger adhesion of the electrode to the diamond, resulting in greater stability. Zhang et al. [77] designed Si-based diamond solar-blind UV detectors utilizing graphite as an electrode. They employed photoresist pyrolysis to prepare the graphite electrode for the diamond film and implemented a planar fork-finger electrode configuration within an MSM structure. Performance testing yielded rise and fall times of 30 ms and 430 ms, respectively. The device also exhibited stable switching characteristics under a 5 V bias voltage, supporting its applicability to UV detection.

### 3.5. Dark-Matter Detection

In recent years, CVD diamond has also been investigated as a potential detector material for low-mass dark-matter searches. In 2019, Kurinsky et al. [78] utilized high-quality CVD diamond to detect dark matter at sub-giga-electron-volt levels. They observed that diamond detectors, compared to conventional semiconductor materials such as Si and Ge, offer higher purity, a lighter carbon nucleus, and a lower dark count rate. These characteristics enhance their sensitivity to electrons and nuclear recoil signals associated with dark matter. The light nuclear mass of carbon facilitates the detection of lower-mass dark matter in nuclear recoil interactions. With further optimization, diamond detectors could significantly expand their application range for detecting low-energy dark matter in the future. In 2022, Abdelhameed et al. [79] experimentally tested two CVD diamond samples equipped with a tungsten film sensor (W-TES) and successfully used diamond as a low-threshold cryogenic detector for sub-GeV dark matter searches, achieving a detection threshold as low as 16.8 eV. Despite these promising results, dark-matter detection represents a substantially different application scenario from conventional space-radiation monitoring. The proposed high-purity diamond concept is primarily intended for detecting very low-energy nuclear and electronic recoils from sub-GeV dark matter [78]. The cryogenic CVD diamond detector reported in [79] operated at approximately 25 mK, highlighting the specialized thermal and readout requirements of this detection approach. Such performance demonstrates the potential of CVD diamond for ultra-low-threshold detection; however, the cryogenic operating temperature, specialized readout architecture, and low-background requirements differ markedly from those of conventional spaceborne radiation monitors. Therefore, dark-matter detection should be regarded here as an emerging application that illustrates the broader sensing capability of CVD diamond rather than as evidence of a mature technology for routine space-radiation monitoring. To provide a comprehensive overview of the application potential of CVD diamond detectors in space environments, Table 5 summarizes representative detector structures, performance parameters, and advantages for different radiation detection scenarios.

The limitations of CVD diamond detectors can be broadly divided into material-intrinsic, growth/device-related, and system-level factors. At the material level, carrier trapping, defect-related non-uniformity, and trapping-induced polarization or electric-field distortion can reduce charge-collection stability. At the growth and device level, impurity incorporation, crystal defects, non-uniform charge transport, contact degradation, and device-to-device variations can affect reproducibility. Large-area high-quality scCVD diamond also remains relatively expensive and less readily available than mature silicon technologies, while multilayer and three-dimensional architectures introduce additional fabrication complexity.

At the system level, detector performance may also be limited by converter-layer efficiency and self-absorption in neutron detectors, calibration uncertainty, packaging stability, front-end electronic noise, and radiation damage to the readout electronics. Spaceborne implementation further requires stable bias supplies, thermal management, vacuum-compatible packaging, electromagnetic compatibility, and long-term calibration monitoring. Therefore, the practical performance of a diamond-based radiation instrument cannot be evaluated from the diamond sensor alone, but must include the stability and radiation tolerance of contacts, converter layers, packaging, electronics, and calibration systems.

## 4. Conclusions and Perspectives

CVD diamond detectors combine high radiation tolerance, low leakage current, fast carrier transport, and excellent thermal robustness, making them particularly attractive for radiation monitoring in harsh space environments. The comparisons presented in this review also show, however, that diamond does not outperform conventional semiconductor detectors in every respect. Silicon remains more mature in large-area fabrication, cost control, standardized packaging, and readout integration, while GaAs and GaN retain advantages in specific detection and high-temperature applications. Therefore, the suitability of CVD diamond should be evaluated according to the radiation environment, mission duration, sensitive area, operating temperature, and system-level constraints of the target mission.

Future development should prioritize measurable improvements at the material, device, and system levels. At the material level, key metrics should include detector-grade crystal area, defect and impurity concentrations, wafer-scale uniformity, carrier lifetime, and charge-collection efficiency. At the device level, performance should be evaluated using leakage current, operating voltage, energy resolution, device-to-device variation, and the stability of electrical contacts. At the system level, quantitative assessment should include readout noise, calibration drift, thermal-vacuum stability, packaging integrity, power consumption, and long-term measurement reproducibility.

Equally important is qualification under realistic space conditions. Radiation tolerance should be reported using clearly specified particle species, energies, accumulated fluence or dose, together with pre- and post-irradiation changes in charge-collection efficiency, leakage current, energy resolution, and calibration response. Environmental qualification should further quantify performance changes after thermal cycling, vacuum exposure, and long-duration operation. In-orbit demonstrations should report mission duration, calibration stability, detector response drift, and instrument-level degradation so that changes originating from the diamond sensor can be distinguished from those associated with contacts, packaging, optical components, and readout electronics. Consequently, CVD diamond should be regarded not as a universal replacement for conventional semiconductor detectors, but as a particularly competitive technology for missions in which radiation tolerance, low dark current, environmental stability, and long operational lifetime outweigh the constraints associated with cost, detector area, and fabrication maturity.

## Figures and Tables

**Table 1:** Key properties of diamond and other semiconductor materials [13,15,16].

	Diamond	Si	GaAs	GaN
Atomic number	6	14	31, 33	31, 7
Mass density/(g/cm^3^)	3.5	2.33	5.32	6.1
Bandgap/eV	5.5	1.1	1.4	3.4
Breakdown strength/(MV/cm)	>10	0.3	0.4	3.4
Mobility/(cm^2^/V·s)	4500 (e) 3800 (h)	1400 (e) 450 (h)	8500 (e) 400 (h)	1200 (e) 200 (h)
Dielectric constant	5.7	11.7	13.1	9.0
Mean energy for e-h pair creation/eV	13.1	3.62	4.3	8.9
Thermal conductivity/(W/cm·K)	22	1.5	0.55	2.1

**Table 2:** Quantitative comparison of representative diamond, Si, GaAs, and GaN radiation detectors.

Parameter	Diamond	Si	GaAs	GaN
Radiation tolerance	SNR ≈ 24 after 1.8 × 10^16^ p·cm^−2^ [17]	Full CCE up to 3 × 10^15^ n_eq_·cm^−2^; ~50% at 10^16^ n_eq_·cm^−2^ [18]	Device-dependent; space-oriented prototypes tested under harsh conditions [19,20]	CCE: 53% → 26% after 10^16^ p·cm^−2^; → 20% after 10^16^ n·cm^−2^ [21,22,23]
Detection performance	scCVD pixel efficiency 99.992 ± 0.002% [17]	~100% CCE at 1.6 × 10^15^ n_eq_·cm^−2^ [18]	Electron efficiency up to 73% at ~70 keV [19]	Maximum CCE 53% before irradiation [23]
Leakage/dark current	1–2 pA at 5 V; PIN ~10^−14^ A [23,24]	Increases with irradiation fluence [18]	1.3 pA at 20 °C → 1.17 nA at 100 °C	1.2 nA at 290 K → 13.9 nA at 450 K [23]
Space-related validation	LYRA/PROBA-2: ~5 yr in orbit; PIN ΔDC ≈ +0.2 ± 0.2% [24]	Extensive flight heritage	Space-oriented detector prototype	Primarily laboratory/prototype detector studies [25]
Main limitation	Cost, limited large-area availability, and device uniformity [26]	Radiation-induced leakage; cooling/high bias at extreme fluence	Active-layer thickness and fabrication complexity	CCE degradation and detector maturity

**Table 3:** Representative characteristics and detector-relevant considerations of major CVD techniques for diamond growth [31,32,33,34,35,37,38,39,40].

Preparation Method	HFCVD	DC-PACVD	DC Arc Jet CVD	MPCVD
Excitation source	Heated filament	DC glow discharge	DC arc plasma jet	Microwave plasma
Growth rate/(μm/h)	1~10	6~25	5~930	0.1~100
Representative deposition scale	Large-area film deposition feasible	Large-area film growth feasible	Large-area and thick-film deposition feasible	Multi-inch or wafer-scale growth demonstrated
Main advantages	Simple equipment, low cost, economical diamond production	Simple equipment, low cost, capability for large-area growth, high growth rate	High growth rate, high diamond quality	Stable equipment, large-area growth, high diamond quality, controllable deposition parameters
Main limitations	Filament contamination, defect control, poor quality, contamination risk	Poor growth quality, contamination risk	Poor equipment stability, electrode contamination	Expensive equipment, optimization required for chambers, low deposition rates
Main detector-relevant impact	More trapping/defects	Uniformity affected	Defect/stress control needed	Favorable carrier transport

**Table 4:** Relative change in dark current (ΔDC) during orbit [75].

Unit-Channel	Detector Type	Bandgap/(FWHM/nm)	ΔDC (47 °C)/%	Cumulative Exposure to Solar Radiation/Days
1-1	MSM diam.	116–126	+4.6 ± 5	7
1-2	PIN diam.	197–237	+0.2 ± 0.2	7
1-3	MSM diam.	17–40 (Al)	+4.0 ± 2	7
1-4	Si-AXUV	6–17 (Zr)	+2.0 ± 2	7
2-1	MSM diam.	116–126	−32 ± 5	1506
2-2	PIN diam.	197–237	+0.2 ± 0.2	1506
2-3	MSM diam.	17–40	−10 ± 2	1506
2-4	MSM diam.	6–17	−26 ± 5	1506
3-1	Si-AXUV	116–126	+14 ± 2	81
3-2	PIN diam.	197–237	+0.2 ± 0.2	81
3-3	Si-AXUV	17–40	+13.5 ± 2	81
3-4	Si-AXUV	6–17	+14 ± 2	81

**Table 5:** Summary of CVD diamond detectors for different space-related applications [24,73,74,75,76,77].

Application	Detector Structure	Radiation Type	Representative Performance	Advantages
Charged particles	MIM, layered, 3D diamond detector	Protons, electrons, heavy ions	High charge collection efficiency (>90%); fast response; radiation tolerance up to high fluence	High radiation hardness, fast timing capability
Neutrons	Diamond PIN detector + ^6^LiF/^10^B converter	Thermal and fast neutrons	Detection efficiency several % with converter layer; stable operation under high neutron fluence	Radiation resistance, compact structure
UV/VUV	PIN/MSM diamond photodiode	Solar UV/VUV	Low dark current; solar-blind response; long-term orbital stability	High temperature stability, radiation tolerance
Dark matter	CVD diamond cryogenic detector	Nuclear/electron recoil	Low threshold detection down to eV level	Low background, high purity material

## Data Availability

No new data were created or analyzed in this study. Data sharing is not applicable to this article.
